# Post–intensive care sequelae after severe bacterial infections in previously healthy children

**DOI:** 10.1186/s40348-026-00245-0

**Published:** 2026-06-01

**Authors:** S. C. Goretzki, C. Held, S. Tschirner, C. Pentek, H. Kölbel, A. Della Marina, A. Gangfuß, H. Reusch, N. Schlaghecke, S. Benson, U. Schara-Schmidt, U. Felderhoff-Müser, C. Dohna-Schwake, N. Bruns

**Affiliations:** 1 Department of Pediatrics I, Neonatology, Pediatric Intensive Care, Pediatric Infectiology, Pediatric Neurology, University Hospital Essen, University, Duisburg-Essen, Hufelandstr. 55, 45147, Essen, Germany; 2 West German Centre for Infectious Diseases (WZI), University Hospital Essen, University Duisburg-Essen, Essen, Germany; 3 Department of Pediatric Neurology, Centre for Neuromuscular Disorders, Centre for Translational Neuro- and Behavioral Sciences, University Duisburg-Essen, Essen, Germany; 4 Institute of Medical Psychology and Behavioral Immunobiology, University Hospital Essen, University of Duisburg-Essen, Essen, Germany

**Keywords:** Invasive streptococcal infections, Post–intensive care follow-up, Neurocognitive outcome, Multidisciplinary care, post-intensive care syndrome (PICS-p)

## Abstract

**Background:**

Severe complications of common community-acquired infections can necessitate pediatric intensive care even in previously healthy children. While survival after pediatric intensive care has improved, data on long-term physical, cognitive, and psychosocial outcomes in this population remain limited. This study evaluated 12-month outcomes in previously healthy children admitted to the pediatric intensive care unit for severe infectious complications and describes the implementation of a structured post-intensive care follow-up program at a tertiary care center in Germany.

**Methods:**

We conducted a retrospective single-center study including previously healthy children admitted to the PICU in 2023 due to severe complications of community-acquired infections. Patients participated in a structured multidisciplinary follow-up program with standardized neurological, physical, cognitive, and psychosocial assessments performed up to 12 months after discharge.

**Results:**

Thirty-seven previously healthy children admitted in 2023 were included. The largest subgroup of the study population consisted of children with intracranial abscess or meningitis (43%), orbital abscess (27%), septic shock (14%), osteomyelitis (11%), and necrotizing fasciitis (5%). Despite appropriate acute management and survival of all patients, 25 children (68%) developed persistent neurological, somatic, or psychosocial impairments during follow-up. Common sequelae included motor deficits, neurocognitive dysfunction, sensory impairment, anxiety, and reduced physical endurance. Importantly, many complications were not apparent early after discharge and emerged several months later, with a median time to identification of approximately five months. Recovery trajectories varied considerably, reflecting the multidimensional nature of pediatric post–intensive care morbidity.

**Conclusions:**

A substantial proportion of previously healthy children experience clinically relevant, multidimensional morbidity after severe infections requiring intensive care. These findings highlight the importance of structured, multidisciplinary post-intensive care follow-up to enable early recognition of delayed sequelae and timely intervention, with the potential to reduce long-term morbidity and improve quality of life for affected children and their families.

## Introduction

Advances in pediatric intensive care medicine have led to a substantial decline in mortality rates [1]. As survival improves, attention has shifted toward long-term morbidity and health-related quality of life, with up to 80% of children developing new or persistent physical, cognitive, psychological, or social impairments after discharge [2, 3].

Pediatric post–intensive care syndrome describes newly acquired or worsening impairments after pediatric intensive care, while family members may also be affected [4], highlighting the importance of structured, multidisciplinary follow-up [5].

While most bacterial infections in childhood are benign and self-limiting, life-threatening complications such as sepsis, intracranial abscess, necrotizing fasciitis, or osteomyelitis can arise even in previously healthy children [6–8]. They may experience substantial long-term sequelae after PICU discharge [4, 9].

This brief report describes post-discharge morbidity in children admitted to a single pediatric intensive care unit in 2023 with severe bacterial infectious complications, highlighting PICS-like impairments.

## Methods

**Fig. 1:**
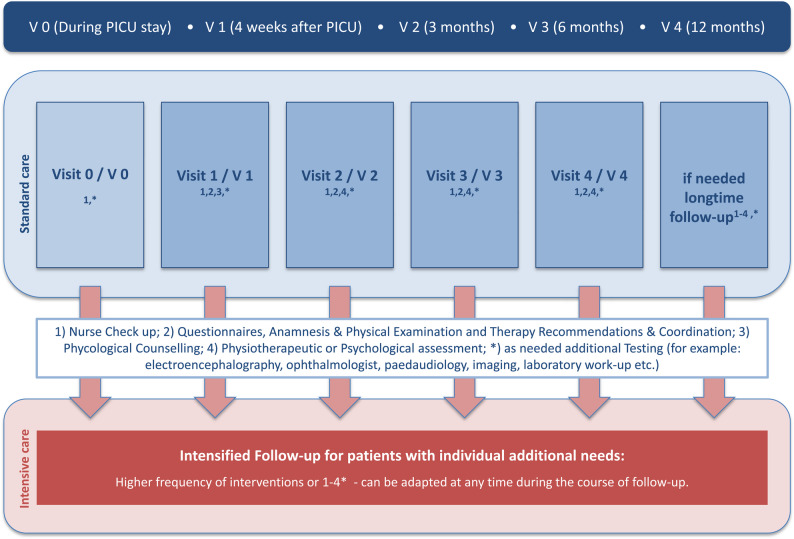
Timeline of structured post-PICU follow-up visits over 12 months

This was a retrospective, single-center study conducted at a tertiary care children’s hospital. Children > 28 days and < 18 years admitted to the PICU in 2023 due to complications of community-acquired ear, nose, and throat (ENT) or soft-tissue infections were included. Anonymized data from a structured post–intensive care follow-up program were analyzed, including key inpatient risk factors (Fig. 1). Multidomain outcomes were assessed at 4 weeks and at 3, 6, and 12 months after discharge using age-adapted standardized physical, neurocognitive, and psychosocial assessments by trained pediatric neurologists, psychologists, physiotherapists, developmental specialists, and pediatricians experienced in pediatric post-intensive care syndrome (PICS-p) (Table 1).

Descriptive analyses were performed; continuous variables are presented as median (interquartile range) and categorical variables as n (%).

Vaccination status and preventive counseling were routinely reviewed during follow-up visits as part of standard pediatric care; however, these data were not systematically collected as predefined study outcomes and were therefore not included in the analysis.

## Results

A total of 37 children fulfilled the inclusion criteria. The median age at admission was 8 years (IQR 1–11.5), and 61% were female. All patients had been previously healthy before PICU admission. The included cohort represented approximately 6% of all annual PICU admissions (including postoperative monitoring and planned admissions) in 2023.

The primary infectious foci were ENT infections (*n* = 27; 73%) and soft-tissue infections (*n* = 10; 27%). The largest clinical subgroup consisted of children with intracranial abscess or bacterial meningitis (*n* = 17; 43%), retro-orbital abscess (*n* = 10; 27%), septic shock (*n* = 5; 14%; including three toxic shock syndromes), osteomyelitis (*n* = 4; 11%), and necrotizing fasciitis (*n* = 1; 5% (Table 1; Fig. 1).

**Table 1:** Patient characteristics, sequalaes and PICU-Follow-Up

Variable		*n* (%) or Median (IQR)
Demographics		
Baseline Characteristics		
Age at admission, years, *median (IQR)*		8 (1–11,5)
Female		22 (61%)
Previously healthy before infection		37 (100%)
Primary infectious focus		
Ear-Nose-Throat-Infection		27 (73%)
Soft-Tissue-Infection (incl. cellulitis and phlegmone)		10 (27%)
Primary infectious complication leading to PICU admission		
Intracranial abscess / bacterial meningitis		17 (43%)
Retro-orbital abscess		10 (27%)
Septic shock (incl. toxic shock syndrome)		5 (14%)
Osteomyelitis		4 (11%)
Necrotizing fasciitis		1 (5%)
Pathogens identified		
* Streptococcus* spp.		18 (49%)
* Streptococcus pyogens*		13 (35%)
* Streptococcus pneumoniae*		1 (3%)
* Streptococcus intermedius*		4 (11%)
* Staphylococcus aureus*		9 (24%)
Other bacteria*		10 (27%)
PICU Course		
Antimicrobial therapy****		35 (95%)
Surgical source control (drainage/debridement)		32 (87%)
Length of PICU stay, days, *median (IQR)*		4 (2–5)
Mechanical ventilation		30 (81%)
Length of mechanical ventilation, days, *median (IQR)*		1 (1–2)
Vasoactive support		14 (38%)
Duration of vasoactive support, days, *median (IQR)*		1 (1–3.5)
Sedative medication		30 (81%)
Sedation duration, days, *median (IQR)*		2 (1–4)
Delirium		12 (32%)
Sequelae		
Overall Outcome		
PCPC change (from admission to discharge), *median (IQR)*		1 (0–1)
PCPC before admission, *median (IQR)*		1 (1–1)
PCPC after admission, *median (IQR)*		2 (1–2)
Mortality		0 (0%)
Sequelae		25 (68%)
Time to diagnosis of sequelae, month, *median (IQR)*		5 (1–3)
Specific sequelae		
Seizures (newly diagnosed after discharge)		8 (22%)
Newly diagnosed migraine (needing medication)		10 (27%)
Motor deficits (physiotherapeutic assessment, necessity of aids)		10 (27%)
Visual impairment (ophthalmologically confirmed)		8 (22%)
Hearing impairment (decrease in audiogram)		10 (27%)
Developmental regression or lack of developmental progress (psychological and pediatric assessment using among others *Bayley Scales of Infant Development testing and WISC-V)*		10 (27%)
Attention deficits (needing therapy/medication, school difficulties		8 (22%)
Depression, anxiety (psychological evaluated and need for therapy - previously screened using ILK, BAI-Y, TEX-Q)		9 (24%)
Therapies		
Inpatient rehabilitation		17 (46%)
Referral to psychologist		12 (32%)
Referral to physiotherapy		14 (38%)
Referral to occupational therapy		18 (49%)
Referral to speech therapy		8 (22%)
Medication after discharge		13 (35%)
Others**		7 (19%)
Difficulties within coding resulting funding challenges		
All therapies		22 (59%)
Inpatient rehabilitation		13 / 17 (45%)
Follow-up Assesments		
Follow-up attendence and continuity***		
Attended Post-PICU Visit 1 (≈ 4 weeks)		30 (81%)
Attended Post-PICU Visit 2 (≈ 3 months)		25 (68%)
Attended Post-PICU Visit 3 (≈ 6 months)		23 (62%)
Attended Post-PICU Visit 4 (≈ 12 months)		23 (62%)
Additional visits (between standard visits)		11 (30%)
Overview of the standardized multidomain follow-up test battery**		
Domain and month after Discharge	Assessment Tools / Methods	Purpose / Outcome Measured
Physical/Somatic Function, months: 1, 3, 6, 12	Comprehensive pediatric examination with neuro-pediatric focus; as well as physiotherapeutic assessment	Motor deficits, muscle tone, coordination, residual neurological impairment, pain, developmental state
Neurocognitive Development, months: 3, 6, 12	*Bayley Scales of Infant and Toddler Development* (for < 3 years); *Wechsler Intelligence Scale for Children – V* (WISC-V, for ≥ 6 years)	Global and domain-specific cognitive performance; developmental progress or regression
Psychological/Emotional Function, months: 3, 6, 12	*Beck Anxiety Inventory for Youth* (BAI-Y); *Inventory for the Assessment of Quality of Life in Children and Adolescents* (ILK)	Anxiety symptoms, depression, health-related quality of life
Body Image/Self-Perception, month: 6, 12	*Body Self-Perception Inventory for Youth* (BSCI-Y)	Self-esteem, perception of physical changes after illness
Therapy Motivation/Expectation, months: 3, 12	*Therapy Expectation Questionnaire* (TEX-Q)	Patient and family expectations and satisfaction regarding rehabilitation and follow-up care
Family/ Psychosocial Impact, months: 6, 12	Semi-structured family interviews and psychologist evaluation	Family functioning, parental stress, PICS-F screening

*Others included atypical mycobacteria (*n* = 2), Klebsiella spp. (*n* = 1), mycoplasma (*n* = 1), unknown pathogen (*n* = 6); **Others: plastic issues [4], partial limp loss [2], chronic pain [1], Cochlea implant [1]; *** Reasons for not attendance: still in inpatient rehabilitation [3], no further need for follow-up [4], family moved away [3], incompliance/missed appointments [4]; Abbreviation: BAI-Y = Beck Anxiety Inventory for Youth; BSCI-Y = Body Self-Perception Inventory for Youth; ILK = Quality of Life in Children and Adolescents; PCPC = Pediatric Cerebral Performance Category Scale - Longitudinal recovery trajectories likely vary substantially over time; however, detailed serial Pediatric Cerebral Performance Category (PCPC) assessments were not consistently available for all patients because of the retrospective study design.; PICU = Paediatric Intensive Care Unit; Streptococcus spp. = Streptococcus species; TEX-Q = Therapy Expectation Questionnaire; WISC-V = Wechsler Intelligence Scale for Children-V; ** Assessments were age-adapted and administered by trained pediatric neurologists, psychologists, and physiotherapists. Questionnaires not validated for younger children were either parent-reported or applied only in older children and adolescents. Younger children underwent structured developmental and psychosocial evaluation using neurological examinations, psychologist-guided parental interviews, observational assessments, and validated developmental tools where appropriate. **** Two patients were managed with surgical source control and local antiseptic treatment alone without prolonged systemic targeted antimicrobial therapy

Pathogen detection revealed Streptococcus spp. in 18 (49%), *Staphylococcus aureus* in 9 (24%), and other bacteria in 10 (27%) cases, including atypical Mycobacteria (*n* = 2; 5%), Klebsiella spp. (*n* = 1; 3%), Mycoplasma (*n* = 1, 3%), and six cases without an identified pathogen. Targeted antimicrobial therapy was received by 95% and 32 (87%) required surgical source control (Table 1).

The median PICU stay was 4 days (Interquartile range (IQR) 2–5). Mechanical ventilation was required in 30 (81%) patients for a median of 1 day (IQR 1–2). Vasoactive support was administered in 14 (38%), and sedation was administered in 30 (81%), half of which showed signs of delirium.

All patients survived; long-term sequelae were observed in 25 (68%). The most frequent neurological or functional impairments included motor deficits (27%), hearing impairment (27%), migraine requiring new long-term medication (27%), and visual impairment (22%). Seizures, developmental regression, and attention deficits each occurred in one-quarter of cases (Table 1). The Pediatric Cerebral Performance Category Scale (PCPC) worsened by a median of 1 (IQR 0–1).

Beyond physical impairment, psychological and cognitive difficulties also became evident during follow-up. Depression or anxiety was documented in 9 (24%) children, and 8 (22%) received support for attention or concentration problems, leading to school-related difficulties such as a decline in grades by at least one level, repetition of a school year, transfer to a different type of school, or even the need for an integration assistant. Inpatient rehabilitation was initiated in 17 (46%), and many required outpatient therapies, including occupational therapy (49%), physiotherapy (38%), psychological care (32%), and speech therapy (22%) (Table 1).

**Fig. 2:**
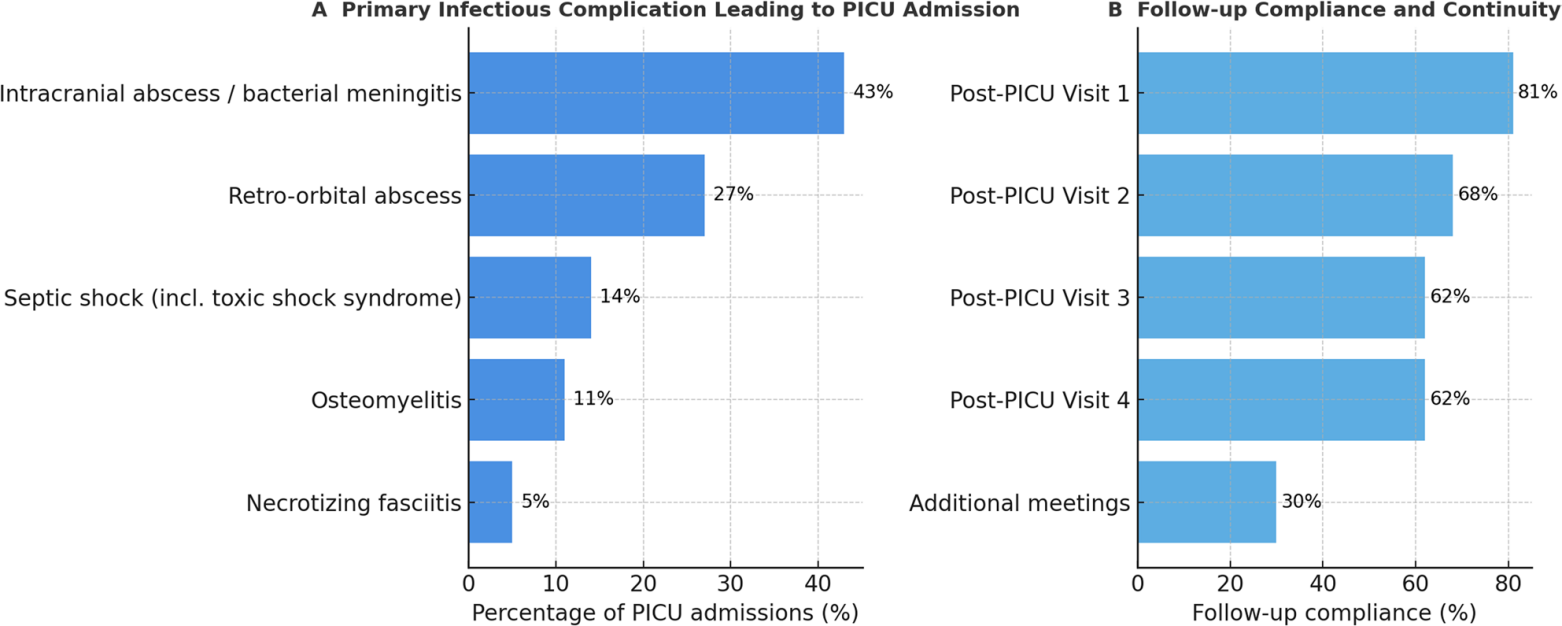
Infectious complications and follow-up attendance (A Infection leading to PICU Admission; B Follow-up Compliance and Continuity)

The attendance at 4 weeks, 3 months, 6 months, and 12 months (Fig. 1) declined from 81% at Visit 1 to 62% after 1 year. The main reasons for missed visits were ongoing inpatient rehabilitation (*n* = 3), no further need for follow-up (*n* = 4), family relocation (*n* = 3), and non-attendance (*n* = 4). Additional follow-up visits were required in 11 (30%) patients to address ongoing issues (Table 1; Fig. 2).

In 22 children (59%), difficulties in diagnostic coding led to delays or denials in reimbursement for required therapies, particularly affecting access to specialized inpatient rehabilitation (13/17; 45%), where the absence of a specific ICD-10 code for PICS-p frequently necessitated additional justification (Table 1; Fig. 2).

## Discussion

This single-center analysis found that initially benign bacterial infections that progress to severe or even life-threatening complications can cause long-term morbidity in previously healthy children.

The predominance of Streptococcus spp. corresponds to the surge of invasive streptococcal infections reported in 2023 across Europe and North America, likely reflecting post-pandemic changes in population immunity and pathogen transmission dynamics [10]. In our cohort, this was particularly reflected by the high proportion of severe ENT-associated invasive complications.

A large proportion of children surviving intensive care develop persistent sequelae despite short PICU stays and often mild organ dysfunction. In this cohort, long-term morbidity likely reflected both the underlying neurological infectious complications, particularly intracranial abscesses and meningitis, as well as critical illness–related morbidity. These include motor and sensory deficits, neurocognitive changes, and psychosocial difficulties, consistent with the multidimensional concept of pediatric Post-Intensive Care Syndrome (PICS-p) [2]. A recent meta-analysis showed reduced cognitive performance and post-traumatic stress even without primary neurological injury [3]. It is assumed that post-critical illness morbidity is common, under-recognized, and influenced by factors such as sedation, delirium, pain, and stress during the PICU stay [11].

Emerging frameworks, like that described by Manning et al., conceptualize PICS-p as encompassing physical, cognitive, and psychosocial domains [12]. Our findings support this model: many psychosocial and cognitive sequelae emerged months after hospital discharge, typically between three and six months, showing limits of early outcome assessments. Similar findings support structured follow-up, yet its implementation remains inconsistent [13].

The structured post-PICU follow-up in our cohort achieved high overall attendance, with 81% attendance at four weeks and 62% at one year, demonstrating its clinical feasibility. Approximately 30% of patients required additional visits between scheduled visits or after the 12-month assessment, underscoring the need for flexible follow-up intervals.

Integrating psychological, physiotherapeutic, and educational support proved crucial for early detection and intervention in our follow-up program. Our data suggest that even children with severe but non-life-threatening illness benefit from systematic follow-up, as subtle morbidity often became apparent only after school or social reintegration. This aligns with international calls to shift the focus from short-term survival to long-term functional and psychosocial outcomes [13]. However, morbidity in this cohort likely reflected both infection severity and critical illness associated factors.

Limitations include the retrospective, single-center design and modest sample size, which limit generalizability. Due to the modest sample size and descriptive design, formal analyses evaluating associations between PICU severity markers, therapeutic interventions (e.g., mechanical ventilation, sedation, delirium), and long-term morbidity were not feasible. Future multicenter prospective studies should systematically investigate these relationships. Moreover, a detailed characterization of this patient population is essential from both a health-services and health-economics perspective. The absence of a dedicated International Classification of Diseases diagnostic code for pediatric post–intensive care morbidity complicates standardized documentation and reimbursement of long-term sequelae [14, 15]. Another important limitation is the lack of uniformly validated pediatric PICS-specific assessment tools across different age groups, highlighting the need for standardized pediatric follow-up instruments. International studies consistently report persistent physical, cognitive, psychosocial, and educational impairments after pediatric critical illness, yet structured follow-up and rehabilitation pathways remain inconsistently implemented [5]. These findings underscore that post–intensive care morbidity extends beyond medical recovery and requires coordinated, multidisciplinary care. Our findings therefore support the development of a multiprofessional post-ICU follow-up model to address these structural gaps and to ensure that children and families receive appropriate, continuous and equitable care across the entire recovery trajectory.

## Conclusion

This single-center analysis demonstrates that structured follow-up for pediatric intensive care survivors is both feasible and valuable. Previously healthy children with severe and partially life-threatening infectious complications may develop relevant long-term neurological, functional, cognitive, and psychosocial sequelae. Multidisciplinary post-PICU care enabled early detection of neurological, functional, and psychosocial impairments and was well accepted. These findings support systematic follow-up to all PICU survivors, while multicenter prospective studies should define optimal screening strategies to identify PICS-p and improve long-term outcomes.

## Data Availability

No datasets were generated or analysed during the current study.
